# A New Trajectory Tracking Algorithm for Autonomous Vehicles Based on Model Predictive Control

**DOI:** 10.3390/s21217165

**Published:** 2021-10-28

**Authors:** Zhejun Huang, Huiyun Li, Wenfei Li, Jia Liu, Chao Huang, Zhiheng Yang, Wenqi Fang

**Affiliations:** 1 Shenzhen Institutes of Advanced Technology, Chinese Academy of Sciences, Shenzhen 518055, China; zj.huang@siat.ac.cn (Z.H.); wf.li1@siat.ac.cn (W.L.); jia.liu1@siat.ac.cn (J.L.); zh.yang@siat.ac.cn (Z.Y.); 2 CAS Key Laboratory of Human-Machine Intelligence-Synergy Systems, Shenzhen Institutes of Advanced Technology, Shenzhen 518055, China; 3 Guangdong-Hong Kong-Macao Joint Laboratory of Human-Machine Intelligence-Synergy Systems, Shenzhen 518055, China; 4 Department of Industrial and Systems Engineering, The Hong Kong Polytechnic University, Hong Kong 999077, China; hchao.huang@polyu.edu.hk; 5 Research Center of Digital Intelligence Technology, Nanhu Lab, Jiaxing 314033, China; wqfang@nanhulab.ac.cn

**Keywords:** autonomous driving, trajectory tracking, real-time control, model predictive control

## Abstract

Trajectory tracking is a key technology for precisely controlling autonomous vehicles. In this paper, we propose a trajectory-tracking method based on model predictive control. Instead of using the forward Euler integration method, the backward Euler integration method is used to establish the predictive model. To meet the real-time requirement, a constraint is imposed on the control law and the warm-start technique is employed. The MPC-based controller is proved to be stable. The simulation results demonstrate that, at the cost of no or a little increase in computational time, the tracking performance of the controller is much better than that of controllers using the forward Euler method. The maximum lateral errors are reduced by 69.09%, 47.89% and 78.66%. The real-time performance of the MPC controller is good. The calculation time is below 0.0203 s, which is shorter than the control period.

## 1. Introduction

**Figure 1:**
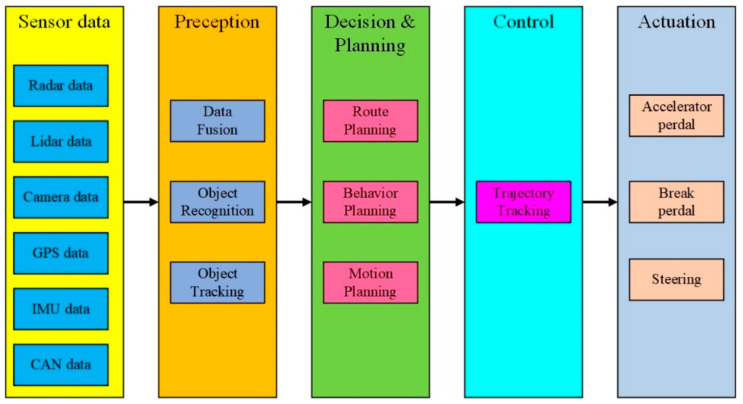
General framework of an autonomous driving system comprising perception, decision and planning, control and actuation.

Research in autonomous driving has aroused increasingly more attention of late [1,2]. The most basic and important goal of an autonomous passenger vehicle is to free people from driving and safely take passengers from an initial state to a final state in a desired interval of time. The architecture of contemporary autonomous driving systems is typically organized into the perception system and the decision-making system [3]. The perception system takes charge of estimating the vehicle states and representing the surrounding environment using data from sensors, including Light Detection and Ranging (LIDAR), Radio Detection and Ranging (RADAR), cameras, a Global Positioning System (GPS), and an Inertial Measurement Unit (IMU). In particular, camera data is of vital importance. Tesla released its fully self-driving version 9 Beta software on 10 July 2021, which relies on camera vision and neural net processing to deliver autopilot. The Lane Support System (LSS) uses cameras to identify the road lines and alert drivers to potential hazards. However, there is still much uncertainty regarding the vision needs of LSS and the results of the experimental tests for LSS are quite limited [4,5]. Cafiso and Pappalardo [4] developed logit models to investigate road characteristics and conditions that affects LSS performance and employed the Firth’s penalized maximum-likelihood method to estimate the logistic regression coefficients and standard errors to describe the rareness of the events. They gave threshold values for the luminance coefficient in diffuse lighting conditions and horizontal curvature radius, and presented remarks on road maintenance and design standards. Pappalardo et al. [5] experimentally tested LSS performance in two lane rural roads with distinct geometric alignments and road marking conditions. They proposed a decision tree method to analyze the cause of the LSS faults and the effects of the variables involved. On the other hand, the decision-making system takes charge of navigating a car from the current position to a goal position safely, feasibly, timely, and comfortably [6]. The decision-making system can be further divided into three subsystems: a decision and planning system, a control system, and an actuation system. Of them, motion planning is a key autonomous driving technique. Li and Shao [7] proposed a motion planner for autonomous parking, and the time-optimal dynamic optimization problem with vehicle kinematics, collision-avoidance conditions and mechanical constraints was solved using a simultaneous approach using the interior-point method. Zhang [8] proposed a hierarchical three-layer trajectory planning framework to realize real-time collision avoidance on highways under complex driving conditions. Therefore, a general framework of an autonomous driving system is shown in Figure 1. Besides a perception and decision-making system, advanced X-by-wire chassis, including drive-by-wire, steer-by-wire, brake-by-wire and active/semi-active suspension subsystems are of vital importance to improving the performance and safety of connected and autonomous vehicles. Zhang et al. [9] proposed a fault-tolerant control method for steer-by-wire systems to mitigate the undesirable influence of front wheel steering angle sensor faults via the use of the Kalman filtering technique. A complete and systematic survey on chassis coordinated control methods for full X-by-wire vehicles can be found in [10]. Here we focus on trajectory tracking, which is a key technology for precisely controlling autonomous vehicles.

The trajectory tracking algorithms are designed to ensure that a vehicle follows a predetermined trajectory generated either offline using navigation systems or online using the motion planning module. The performance of trajectory tracking directly determines the performance of autonomous vehicles, which involves driving safety, passenger comfort, travel efficiency, and energy consumption [11]. The trajectory tracking control of autonomous vehicles is a challenging research area because these systems typically are nonlinear systems with non-holonomic constraints.

Pure-pursuit [12] and the Stanley method [13] are two prevalent geometric controllers. The main advantage of these methods is that they use simple geometric models with few parameters, and therefore can give timely feedback on the current state and constraints to meet the real-time requirement of an autonomous vehicle. The pure-pursuit method and its variants are one of the most commonly used methods to solve the path-tracking problem for mobile robots [14]. The Stanley method is the path-tracking approach used by Stanley, Stanford University’s autonomous car; Stanley won the DARPA Grand Challenge in 2005 [13]. However, these methods have their limitations. Pure-pursuit control works as a proportional controller of the steering angle operating on the cross-track error by calculating the curvature from the current position to some goal position. When the look-ahead distance is too large, its performance is poor, and the vehicle may cut corners when changing direction or making a U-turn. The Stanley method considers both the heading and cross-track errors and therefore it is more effective and steady than the pure-pursuit method. But it does not perform well on discontinuous paths. To sum up, geometric-based tracking controllers (pure pursuit, Stanley, etc.) have a simple structure and are easy to implement. However, they are not suitable for applications that need to consider vehicle dynamics (e.g., high-speed trajectory tracking, extreme path curvature, etc.). It is also difficult to achieve a trade-off between stability and tracking performance [15].

Proportional-Integral-Derivative controllers (PID) [16] and sliding model controllers (SMC) [17] are two prevalent classical control algorithms. Although PID controllers have good tracking performance, there is a major challenge in the tuning of the parameters because of the vehicle and tire nonlinearities. SMC is a well-developed nonlinear state-feedback controller and has been used to design vehicle trajectory tracking controllers. Because of the nonlinear control law, SMC shows good tracking accuracy. However, there are several drawbacks: first, its performance is sensitive to the sampling rate of the controller; second, chattering problems exist under certain conditions [18]; third, robustness is only guaranteed on the sliding surface; and lastly, it needs prior knowledge [19]. To sum up, compared with geometric-based tracking controllers, model-based tracking methods are more feasible and reliable in real driving scenarios at the cost of the increase in computational burden and complexity.

Reinforcement learning (RL) has shown an ability to achieve super-human results at turn-based games like Go [20] and chess [21]. Deep RL has been applied to the decision-making system of autonomous driving in several simulated environments [22]. Mohammadi et al. [23] proposed an optimal tracking controller for nonlinear continuous-time systems with time-delay, mismatched external disturbances, and input constraints, using the technique of integral reinforcement learning and a Hamilton-Jacobi-Bellman equation. However, there are two main limitations for RL-based methods. First, they require large amounts of data to build up a feasible model; specifically, data is sometimes expensive and hard to obtain. Second, they require a sufficiently long time to train the model to complete the specific tasks due to significant data manipulation. The performance of the controllers using machine-learning methods relies on the learning capability of the model and the quality of the data.

**Figure 2:**
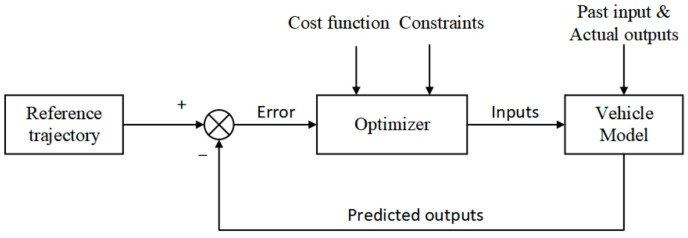
General framework of an MPC structure.

Model Predictive Control (MPC) has been applied to trajectory planning and tracking of an autonomous vehicle due to its flexibility and ability to compute optimal solutions with hard and soft constraints [24,25]. Shen et al. [24] proposed a unified receding-horizon optimization scheme for the integrated path-planning and tracking control of an autonomous underwater vehicle using nonlinear MPC techniques. Borrelli et al. [25] proposed a novel approach to autonomous steering systems based on an MPC scheme. The general framework of an MPC structure is shown in Figure 2. However, these MPC-based tracking controllers are feasible only in low-speed scenarios.

The accuracy of the trajectory tracking control can be greatly improved by improving the accuracy of the predictive model. Most researchers have attempted to improve the accuracy of the kinematic or dynamic model to improve the accuracy of the controller. Few people paid attention to the computation errors during the integration process. With the accumulation of the computation errors, the controller could lose its stability or an accident might even result.

In this paper, we propose a new trajectory-tracking algorithm based on MPC. Instead of using the forward Euler integration method, the backward Euler integration method is used to establish the predictive model.

The contributions here can be summarized as follows:

- A trajectory tracking method is proposed based on MPC. Instead of using the forward Euler method, the backward Euler method is used to establish the predictive model. The proposed method is designed to meet the real-time requirement of autonomous vehicles by structuralizing the control law and employing the warm-start strategy.
- Unlike conventional MPC-based controllers, both the acceleration and steer angle are control inputs. The proposed MPC-based controller can automatically adjust the velocity according to the information of the reference trajectory.
- The dynamic regret of the proposed controller is tightly bounded, and the closed-loop controller is proved to be stable.
- The MPC controller using the backward Euler method has a better tracking accuracy in the lateral error, and it is more robust.

This paper is organized as follows. The MPC-based controller of autonomous vehicles is described in Section 2. After that, the stabilizability of the controller is discussed in Section 3. Simulation results are shown in Section 4. Section 5 concludes this paper by summarizing all of the main results.

## 2. Control Design

**Figure 3:**
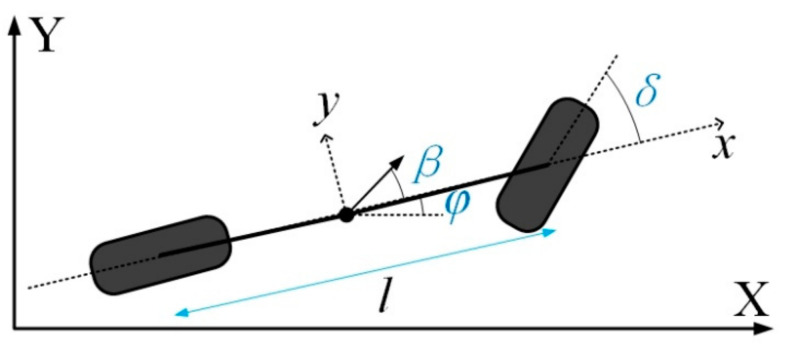
Kinematic rear axle bicycle model of the vehicle.

Establishing a prediction model and designing a rolling optimization function are the kernels of designing a path tracking controller. Due to the strongly nonlinearity of vehicle dynamics, it is very hard to establish a model to describe the actual vehicle dynamics. Researchers generally use Ackermann steering geometry and its simplified bicycle models to describe the vehicle kinematics and dynamics. MPC schemes using dynamic vehicle models and various tire models are generally computationally expensive, and tire models may become singular at low speeds [26]. Kong et al. [26] compared a kinematic and a dynamic bicycle model, and showed that both models could correctly predict a vehicle’s future states, and combining MPC schemes with a simple kinematic bicycle model is less computationally expensive. Polack et al. [27] compared a 3-DOF kinematic bicycle model with a 9-DOF model, and showed that the 3-DOF model could capture enough of the non-holonomic constraints of the actual vehicle dynamics. When the maximum-allowed lateral acceleration of a vehicle was no greater than 0.5 *g* m/s^2^, where *g* is the acceleration due to gravity, using a 3-DOF kinematic bicycle model produces acceptable results and could generate a feasible track. Chen et al. [28] implemented an MPC-based controller for path-tracking using three vehicle dynamics models: a bicycle model, an 8-DOF model and a 14-DOF model. They showed that the bicycle controller could successfully navigate a vehicle along the given path and calculate the optimal steering sequences faster than the controllers with the 8-DOF and 14-DOF vehicle. They concluded that the bicycle controller is suitable for a possible physical implementation with real-time requirements. Therefore, in this paper, the kinematic model of autonomous vehicles is used [26,29]

(1) $$\begin{array}{l} \dot{x}=v\cos(\varphi +\beta ),\\ \dot{y}=v\sin(\varphi +\beta ),\\ \dot{\varphi }=\frac{v\sin(\beta )}{l_{r}},\\ \dot{v}=a,\\ \beta =\tan^{-1}(\frac{l_{r}}{l_{f}+l_{r}}\tan(\delta )), \end{array}$$

where *x* and *y* are the coordinates of the center of mass in an inertial frame (*X*,*Y*). *φ* is the inertial heading, and *v* is the longitudinal speed of the vehicle. The parameters *l_f_* and *l_r_* are the distance from the center to the front and rear axles, and *δ* is the front steering angle. Two front and two rear wheels of the vehicle are combined into single wheels located at the center of the front and rear axle, respectively, as illustrated in Figure 3. *β* is the slip angle at the center of gravity.

In our problem, X = [*x*, *y*, *φ*, *v*] is the vehicle state, U = [*a*, *δ*] is the control state. The model is established based on the following assumptions.

- The vehicle is traveling on a flat surface, with the vehicle’s movement perpendicular to the road surface ignored.
- Only the front wheel can be steered.
- The wind resistance and ground-side friction that the wheels are subjected to while driving are ignored.
- The wheels always maintain good rolling contact with the ground.
- The impact of the vehicle suspension is not taken into account.
- Load transfer is not considered.

The state-space equations of the vehicle system (1) are continuous in time and cannot be used for the design of the MPC algorithm directly. Therefore, the model of the system was converted to discrete state-space equations by discretizing the state-space equations. We assume that the model can be rewritten as

(2) $$\dot{X}=f(X,U).$$

Generally, the state at *k* + 1 instant at time *t* is computed using the forward Euler integration method

(3) $$X(k+1\left|t\right.)=X(k\left|t\right.)+T_{s}\dot{X}(k\left|t\right.)=X(k\left|t\right.)+T_{s}f(X(k\left|t\right.),U(k\left|t\right.)),$$

where *T_s_* is the sampling time.

In this paper, instead of the forward Euler method, the backward Euler method is used to establish the predictive model. Although it requires an extra computation at each iteration, the backward Euler method has great stability properties and its local truncation error is of order $O(T_{s}^{3})$, which is much smaller than $O(T_{s}^{2})$ using the forward Euler method. Hence, the backward Euler method’s error generally decreases faster as $T_{s}\to 0.$

The state at *k* + 1 instant at time *t* is computed using the backward Euler method

(4) $$\begin{array}{l} \tilde{X}(k+1\left|t\right.)=X(k\left|t\right.)+T_{s}f(X(k\left|t\right.),U(k\left|t\right.)),\\ X(k+1\left|t\right.)=X(k\left|t\right.)+T_{s}f(\tilde{X}(k+1\left|t\right.),U(k\left|t\right.)), \end{array}$$

Equation system (8) can be rewritten as

(5) $$\begin{array}{l} X(k+1\left|t\right.)=X(k\left|t\right.)+T_{s}\tilde{f}(X(k\left|t\right.),U(k\left|t\right.)),\\ \tilde{f}(X(k\left|t\right.),U(k\left|t\right.))=f(X(k\left|t\right.)+T_{s}f(X(k\left|t\right.),U(k\left|t\right.)),U(k\left|t\right.)). \end{array}$$

Therefore, the state information of vehicles in the prediction horizon *N_P_* can be obtained

(6) $$\begin{array}{c} X(k+1\left|t\right.)=X(k\left|t\right.)+T_{s}\tilde{f}(X(k\left|t\right.),U(k\left|t\right.)),\\ \vdots \\ X(k+i\left|t\right.)=X(k+i-1\left|t\right.)+T_{s}\tilde{f}(X(k+i-1\left|t\right.),U(k+i-1\left|t\right.)),\\ \vdots \\ X(k+N_{c}+1\left|t\right.)=X(k+N_{c}\left|t\right.)+T_{s}\tilde{f}(X(k+N_{c}\left|t\right.),U(k+N_{c}\left|t\right.)),\\ \vdots \\ X(k+N_{P}\left|t\right.)=X(k+N_{P}-1\left|t\right.)+T_{s}\tilde{f}(X(k+N_{P}-1\left|t\right.),U(k+N_{c}\left|t\right.)), \end{array}$$

where *N_c_* is the control horizon and 1 ≤ *N_c_* ≤ *N_P_*, which denotes component-wise inequality.

The differences between the predictive states and the reference trajectory X*_ref_* are defined as follows

(7) $$\begin{array}{c} e(k+1\left|t\right.)=X(k+1\left|t\right.)-X_{ref}(k+1\left|t\right.),\\ \vdots \\ e(k+N_{P}\left|t\right.)=X(k+N_{P}\left|t\right.)-X_{ref}(k+N_{P}\left|t\right.). \end{array}$$

To ensure the passenger comfort and feasibility of the vehicle, the output control should be varied as smoothly as possible. Therefore, the optimization objective function is defined as

(8) $$J(e(t),U(t))=\sum_{i=1}^{N_{P}}{\left\|e(k+i\left|t\right.)\right\|}_{Q}^{2}+\sum_{i=1}^{N_{c}}{\left\|U(k+i\left|t\right.)-U(k+i-1\left|t\right.)\right\|}_{R}^{2},$$

where *Q* and *R* are the weight matrices for the vehicle states and control states, respectively. Consequently, the rolling optimization can be obtained by solving the constrained optimization problem in every sampling period

(9) $$\begin{array}{l} \underset{U(t)}{\min}J(e(t),U(t))\\ s.t.\;\\ a_{\min}\leq a(k+i\left|t\right.)\leq a_{\max},\;i=1,2,\cdots ,N_{c},\\ \delta_{\min}\leq \delta (k+i\left|t\right.)\leq \delta_{\max},\;i=1,2,\cdots ,N_{c},\\ e_{\min}\leq e(t)\leq e_{\max},\;t=k+T_{s},\cdots ,k+N_{P}T_{s}, \end{array}$$

where (*a*_min_, *a*_max_) and (*δ*_min_, *δ*_max_) are the hard constraints of the vehicle. The last constraints are added to ensure safety driving.

The control inputs are obtained by solving the optimization problem (9). The first element in the control inputs is taken as the optimal control at the current time. After the prediction and control of the current time step are completed, the states are updated with the actual ones, which are then used as the initial states for the optimization problem in the next predictive horizon. The process is repeated until the vehicle reaches the final state.

The problem (9) is a quadratic programming (QP) one which is a traditional optimization problem for trajectory tracking. The first term in the cost function requires that the actual trajectory be as close as possible to the reference trajectory to ensure the safety and feasibility of the trajectory. The second term requires that the control input be varied smoothly to ensure the feasibility of the vehicle and the comfort of passengers. The difference between the reference and the actual trajectory must be sufficiently small. Otherwise, it may lead to a crash, and the trajectory is no longer feasible.

To meet the real-time requirement, instead of directly calculating a control sequence by solving (9), we solve an approximate optimization problem by imposing the constraints *u_k+_*_1_ = *u_k+_*_2_ = *…* = *u_k+Nc_* on the control law. Therefore, we only need to calculate a ‘mediocre’ control to follow the given trajectory. This significantly reduces the complexity of the primal problem as it dramatically reduces the number of the variables. It is worth noting that imposing the constraint conditions *u_k+_*_1_ *= … =* *u_k+Nc_* on (9) is equivalent to setting *N_c_* to 1. Besides greatly reducing the computational burden, one of the most telling advantages of structuralizing the control is to produce an improvement in the robustness and in the general behavior of the system, because allowing the free evolution of the manipulated variables could lead to undesirable high-frequency control signals and even to instability as noted in [30]. We note that, if the coefficient matrices *Q* and *R* are positive semi-definite, the primal problem is tightly bounded and the approximate problem is also tightly bounded. Since *Q* and *R* are positive semi-definite, *x*_1_*^T^Qx*_1_ ≥ 0, *x*_2_*^T^Rx*_2_ ≥ 0. Hence, the cost function in (9) is convex. The feasible region subjected to the constraint conditions (linear equations and inequalities) in (9) is also convex. Thus, the optimal solution of (9) is located in either the interior or the boundary of the feasible region. Therefore, the value of the cost function does not go to infinity, and the primal problem is tightly bounded. When imposing the constraint condition *u_k+_*_1_ *= … = u_k+Nc_*, the corresponding feasible region is still convex since the intersection of convex sets is still a convex set. Similarly, the approximate problem is tightly bounded. In the next section, we prove that the proposed close-loop MPC controller is stable if *N_c_ = N*_1_ = 1, *λ = 0* and *N_P_* is large.

## 3. Stabilizability of Controller

Combining (1) and (5) leads to


(10) $$\begin{array}{l} x_{k+1}=x_{k}+T_{s}(v_{k}+aT_{s})\cos(\varphi_{k}+T_{s}v_{k}\sin(\beta )/l_{r}+\beta ),\\ y_{k+1}=y_{k}+T_{s}(v_{k}+aT_{s})\sin(\varphi_{k}+T_{s}v_{k}\sin(\beta )/l_{r}+\beta ),\\ \varphi_{k+1}=\varphi_{k}+T_{s}(v_{k}+aT_{s})\sin(\beta )/l_{r},\\ v_{k+1}=v_{k}+aT_{s}. \end{array}$$

Equation system (10) can be rewritten as

(11) $$\begin{array}{c} X_{k+1}=\left(I+A_{k}T_{s}\right)X_{k}+B_{k}T_{s}U_{k},\\ A_{k}=\left[\begin{array}{cccc} 0 & 0 & -\left(v_{k}+aT_{s}\right)\sin(\gamma ) & \cos(\gamma )-\left(v_{k}+aT_{s}\right)T_{s}\sin(\gamma )\sin(\beta )/l_{r}\\ 0 & 0 & \left(v_{k}+aT_{s}\right)\cos(\gamma ) & \sin(\gamma )+\left(v_{k}+aT_{s}\right)T_{s}\cos(\gamma )\sin(\beta )/l_{r}\\ 0 & 0 & 0 & \sin(\beta )/l_{r}\\ 0 & 0 & 0 & 0 \end{array}\right],\\ B_{k}=\left[\begin{array}{cc} T_{s}\cos(\gamma ) & -\left(v_{k}+aT_{s}\right)\sin(\gamma )\left(1+v_{k}T_{s}\cos(\beta \right)/l_{r})\beta_{\delta }\\ T_{s}\sin(\gamma ) & \left(v_{k}+aT_{s}\right)\cos(\gamma )\left(1+v_{k}T_{s}\cos(\beta \right)/l_{r})\beta_{\delta }\\ T_{s}\sin(\beta )/l_{r} & \left(v_{k}+aT_{s}\right)\cos(\beta )\beta_{\delta }/l_{r}\\ 1 & 0 \end{array}\right],\\ \gamma =\varphi_{k}+T_{s}v_{k}\sin(\beta )/l_{r}+\beta ,\;\beta_{\delta }=\frac{l_{r}l}{l^{2}\cos^{2}(\delta )+l_{r}^{2}\sin^{2}(\delta )},\;l=l_{f}+l_{r}. \end{array}$$

For the sake of convenience, we omit the subscript *k* in the remainder of this paper.

**Theorem** **1.** *System (11) is controllable*.

**Proof** **of** **Theorem** **1.** First, we seek the eigenvalues *λ* of *A*. By solving the characteristic polynomial det(*λI-A*) = 0, we have

(12) $$\lambda_{1}=\lambda_{2}=\lambda_{3}=\lambda_{4}=0.$$

According to the definition of controllability proposed by Hautus [31], system (4) is controllable if and only if, for all *λ_i_*, *i* = 1, 2, 3, 4 Rank([*λ_i_I-A*,*B*]) = 4. Here we only need to consider Rank([*λ*_1_*I*-*A*,*B*]) due to (12)

(13) $$[\lambda_{1}I-A,B]=\left[\begin{array}{cccccc} 0 & 0 & -A_{13} & -A_{14} & B_{11} & B_{12}\\ 0 & 0 & -A_{23} & -A_{24} & B_{21} & B_{22}\\ 0 & 0 & -A_{33} & -A_{34} & B_{31} & B_{32}\\ 0 & 0 & -A_{43} & -A_{44} & B_{41} & B_{42} \end{array}\right]=[0_{4\times 2},\Omega_{4\times 4}].$$

The determinant of Ω is

(14) $$\det(\Omega )=\frac{{\left(v_{k}+aT_{s}\right)}^{2}l\cos(\beta )}{l^{2}\cos^{2}(\delta )+l_{r}^{2}\sin^{2}(\delta )}\neq 0.$$

Hence we have Rank(Ω) = 4 and 4 ≥ Rank([*λ*_1_*I-A*,*B*]) ≥ Rank(Ω) = 4. □

**Theorem** **2.** *System (11) is observable*.

**Proof** **of** **Theorem** **2.** According to the definition of observability proposed by Hautus [31], system (4) is observable if and only if, for all *λ_i_*, *i* = 1, 2, 3, 4 Rank([*λ_i_I*-*A*;*C*]) = 4.

In our problem, the output function is *Y = X = CX*, and thus *C* = *I*_4__×4_ and Rank(*C*) = 4. Therefore, 4 ≥ Rank([*λ*_1_*I*-*A*;*C*]) ≥ Rank(*C*) = 4. □

**Theorem** **3.** *System (11) is stabilizable*.

**Proof** **of** **Theorem** **3.** According to the definition of stabilizability proposed by Hautus [31], system (11) is stabilizable if and only if *λ_i_* ≥ 0, *i* = 1, 2, 3, 4, and the system is controllable. Combining Theorem 1 and (12) proves that Theorem 3 holds. □

**Theorem** **4.** *The closed-loop MPC controller is stable for N_c_ = 1, λ = 0 and large N_P_.*

**Proof** **of** **Theorem** **4.** This proof is similar as that for Theorem 4 in [32] for generalized predictive control. When *N_P_* is sufficiently large, we have

(15) $$G^{T}G>0,$$

where

(16) $$G={\left[B;AB;\cdots ;A^{N_{P}-1}B\right]}_{N_{P}\times N_{c}}.$$

Therefore, *G^T^G* is a positive scalar, which is always invertible. Therefore, the matrix *G^T^G + λI* is invertible, and a feasible control can be obtained using the expression in [32]

(17) $$u_{opt}={\left(G^{T}G+\lambda I\right)}^{-1}G^{T}(X_{r}-X).$$

Since our optimization problem is convex, there is only one optimal solution and thus our controller will asymptotically converge to (17). □

## 4. Simulation

The simulation environment is MATLAB/Simulink R2020a, and (9) is solved using ‘fmincon’, a built-in function in MATLAB. Sequential quadratic programming is used as the nonlinear solver. The warm-start technique is employed by using the result of the previous optimization problem as a guess for the current optimization problem to further speed up the efficiency of the nonlinear solver. The accuracy of ‘fmincon’ is set to 10^−6^. The processor used in the simulation is Intel(R) Core(TM) i7-4510U @ 2.00 GHz 2.6 GHz. Real-Time Synchronization is enabled to test the real-time performance of the controllers. The simulation system consists of a kinematic model of autonomous vehicles and the trajectory tracking controller proposed in this paper. The parameters of the vehicle model and the controller are shown in Table 1 and can be found in [33]. The road conditions are assumed to be dry and clean and they can support the forces required for braking, accelerating and steering.

### 4.1. Sinusodial Path Following

First, we present the tracking results of a sinusoidal trajectory with an amplitude of 4 m and a wavelength of 100 m in [20]. The reference speed along *x*-axis *Vref* is set to be a constant. The open-loop reference trajectory is given by

(18) Yref=4sin(2πXref/100).

**Figure 4:**
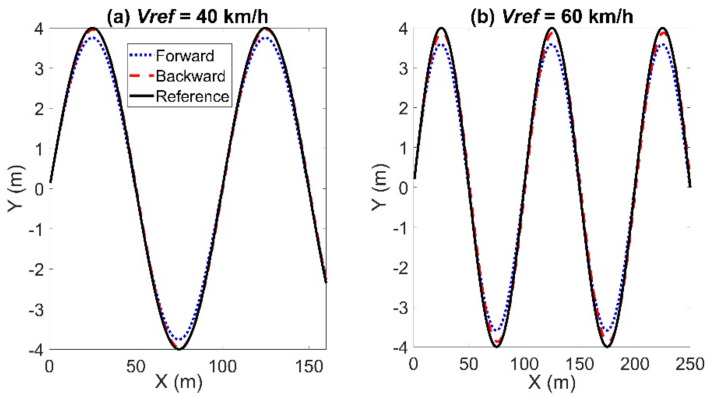
Results for tracking the sinusoidal trajectory with *T_s_* = 0.05 s: left for Case (**a**) with *Vref* = 40 km/h and right for Case (**b**) with *Vref* = 60 km/h.

The tracking result of the sinusoidal trajectory is shown in Figure 4. The sampling time is set to *T_s_* = 0.05 s. The reference trajectory was indicated by the black solid line. The obtained trajectories using the forward and backward Euler method were represented by a blue dotted line and a red dashed line, respectively. When the reference velocity is set to *Vref* = 40 km/h, the maximum lateral error using the backward Euler method was 0.0767 m, in contrast to 0.2481 m using the forward Euler method. The maximum longitudinal errors using the forward and backward Euler method were 0.07 m and 0.0703 m, respectively. The maximum calculation time using the backward Euler method was 0.0203 s, and the average calculation time was 0.0081 s, in contrast to 0.0197 s and 0.01 s using the forward Euler method. The maximum heading errors were 0.0277 rad using the backward Euler method and 0.019 rad using the forward Euler method. When the reference velocity is set to *Vref* = 60 km/h, the maximum lateral error using the Euler method was 0.4191 m; whereas, it was 0.2184 m using the backward Euler method. The maximum calculation times using the forward and backward Euler method were 0.0183 s and 0.0143 s, respectively; the average computation times were 0.0086 s and 0.0084 s; the maximum heading errors were 0.0293 rad and 0.0355 rad. The maximum longitudinal errors are 0.1059 m and 0.1085 m. To sum up, the lateral error using the backward Euler method was much smaller than that using the forward Euler method. However, the longitudinal error and heading error using the backward Euler method were slightly larger than that using the forward Euler method. Besides that, the backward Euler method required a little more calculation time. The state errors, including the lateral, longitudinal and heading errors, increased with the reference velocity.

**Figure 5:**
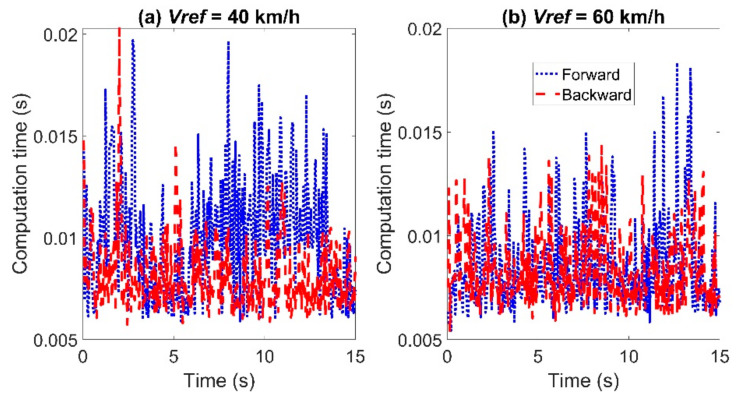
Comparison of the computation time for tracking the sinusoidal trajectory with *T_s_* = 0.05 s: left for Case (**a**) with *Vref* = 40 km/h and right for Case (**b**) with *Vref* = 60 km/h.

The comparison of the calculation time between the two controllers is shown in Figure 5. The calculation time of the MPC controller using the backward Euler method at each control period was almost the same or slightly larger than that of the MPC-based controller using the forward Euler method.

**Figure 6:**
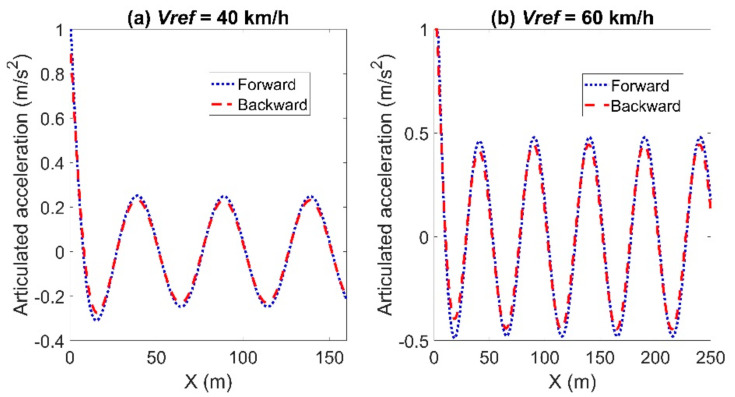
Articulated acceleration for the comparison between the forward and backward Euler method: left for Case (**a**) with *Vref* = 40 km/h and right for Case (**b**) with *Vref* = 60 km/h.

**Figure 7:**
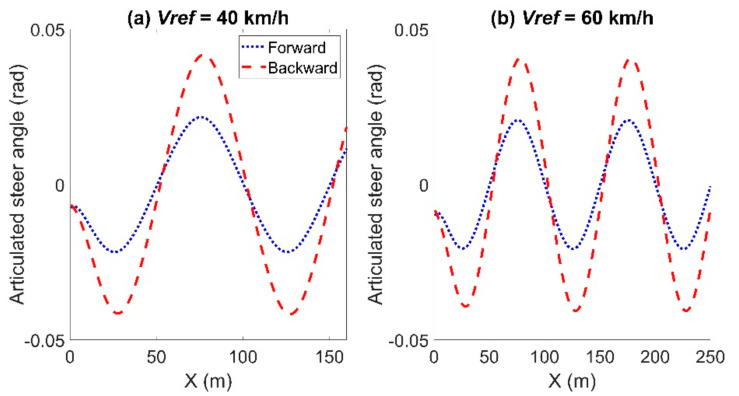
Articulated steer angle for the comparison between the forward and backward Euler method: left for Case (**a**) with *Vref* = 40 km/h and right for Case (**b**) with *Vref* = 60 km/h.

Figure 6 and Figure 7 show the articulated acceleration and steer angle, respectively. The Forward Euler method was more sensitive to the longitudinal velocity, whereas the backward Euler method was more sensitive to the steer angle.

We noted that, as mentioned before, the differences between the reference and actual trajectories increase with the vehicle velocity. There exists a threshold value of velocity to determine the existence of the solution of the optimization problem for trajectory tracking. In other words, when the reference velocity is greater than some value, no feasible solution exists. When *T_s_* = 0.05 s, the threshold value of the reference velocity using the forward Euler method was 67.7 km/h (when *Vref* = 67.8 km/h, the maximum lateral error was 0.5009 m), whereas it was 83 km/h using the backward Euler method (when *Vref* = 83.1 km/h, the maximum lateral error was 0.5002 m). Hence, the MPC using the backward Euler method was more robust than that using the forward Euler method.

### 4.2. Circular Path

In the second scenario, the vehicle was required to track a circle with a radius of 40 m. The parametric equations for the circle were

(19) $$\left\{\begin{array}{l} X(t)=Rd\cos(\varphi (t)-\pi /2),\\ Y(t)=Rd+Rd\sin(\varphi (t)-\pi /2),\\ \varphi (t)=\;t\;Vref/Rd, \end{array}\right.$$

where *Rd* is the radius of the reference circle. The initial configuration and constraint conditions were chosen to be same as previously to be consistent. The sampling time and the reference velocity are set to *T_s_* = 0.05 s and *Vref* = 10 m/s, respectively.

**Figure 8:**
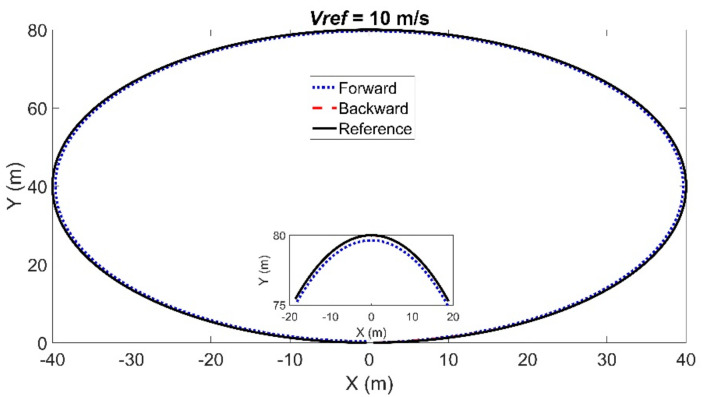
Simulation results for the circular path with radius *Rd* = 40 m. Notations the same as in Figure 4.

The tracking result of the circular path is shown in Figure 8. The maximum lateral and longitudinal errors using the backward Euler method were 0.0596 m and 0.0091 m, in contrast to 0.3664 m and 0.3664 m using the forward Euler method. The maximum calculation time using the backward Euler method was 0.016 s, and the average calculation time was 0.0084 s, in contrast to 0.0199 s and 0.0085 s using the forward Euler method. The maximum heading errors were 0.0411 rad using the backward Euler method and 0.0194 rad using the forward Euler method. In sum, the MPC controller using the backward Euler method had a better tracking accuracy in the circular path than that using the forward Euler method.

**Figure 9:**
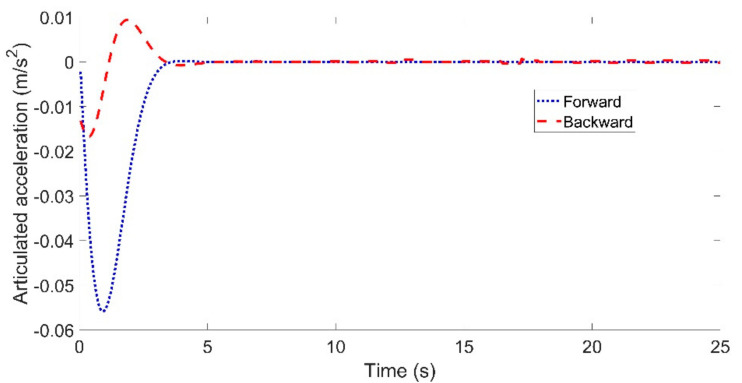
Articulated acceleration for the comparison between the forward and backward Euler method for the circular trajectory.

**Figure 10:**
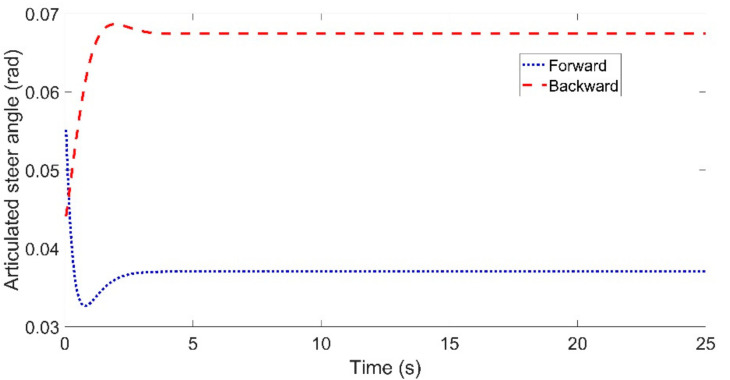
Articulated steer angle for the comparison between the forward and backward Euler method for the circular trajectory.

**Figure 11:**
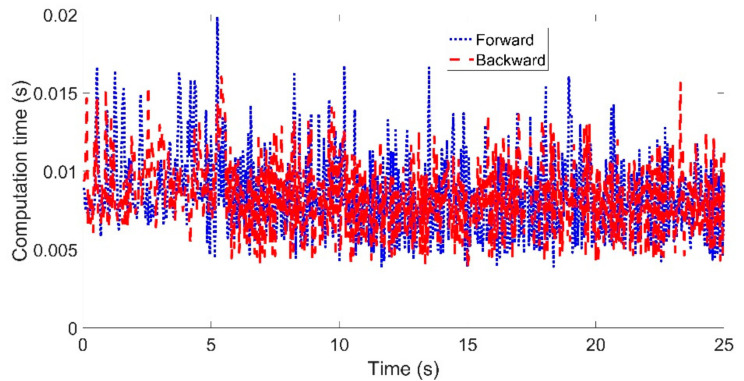
Comparison of the computation time for the circular path. Red dashed curve: MPC-based controller using the backward Euler method; blue dotted curve: MPC-based controller using the forward Euler method.

Figure 9 and Figure 10 show the articulated acceleration and steer angle, respectively. The articulated acceleration and steer angle using the backward Euler method were quite different from those using the forward Euler method. As can be seen from Figure 8, the backward Euler method was more accurate than the forward Euler method, and the calculation times were almost the same as shown in Figure 11.

### 4.3. Double Line Change Path

**Figure 12:**
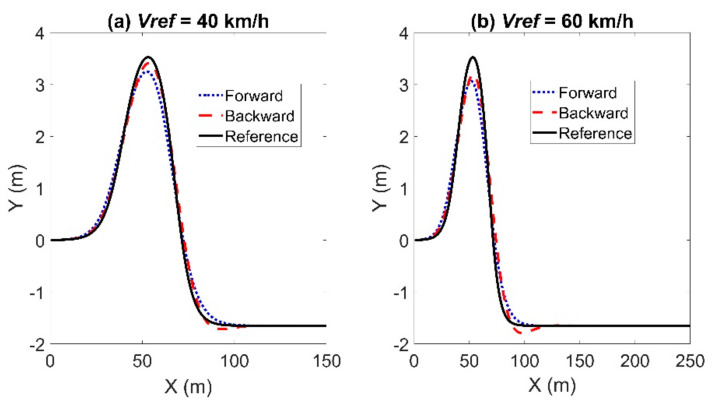
Simulation results for the double line change path: left for Case (**a**) with *Vref* = 40 km/h and right for Case (**b**) with *Vref* = 60 km/h.

**Figure 13:**
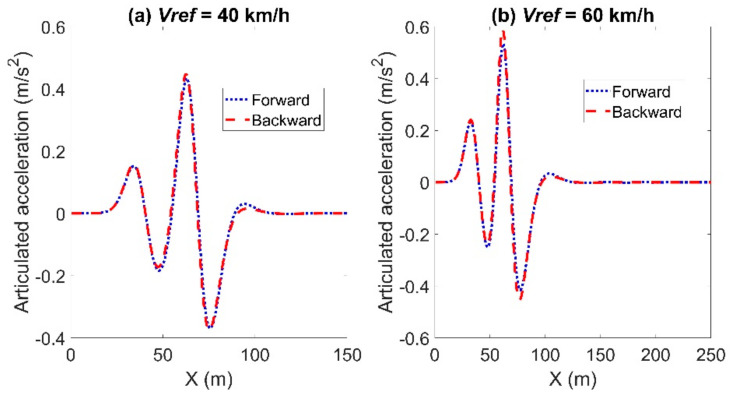
Articulated acceleration for the comparison between the forward and backward Euler method for the circular trajectory: left for Case (**a**) with *Vref* = 40 km/h and right for Case (**b**) with *Vref* = 60 km/h.

**Figure 14:**
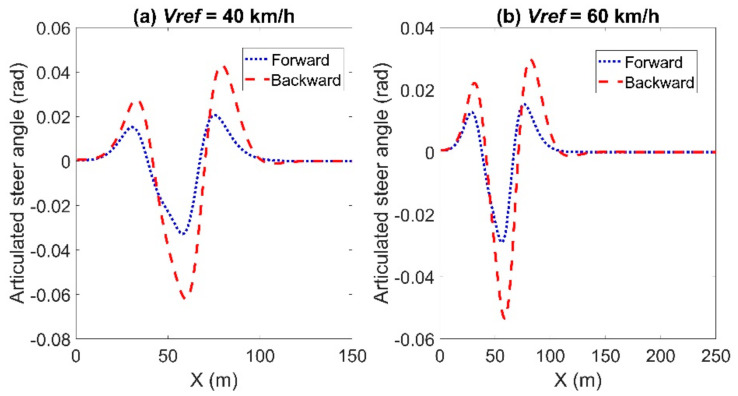
Articulated steer angle for the comparison between the forward and backward Euler method for the circular trajectory: left for Case (**a**) with *Vref* = 40 km/h and right for Case (**b**) with *Vref* = 60 km/h.

In this scenario, the vehicle was required to track a double line change path. The reference trajectory of the double line change path can be found in [33]. The tracking result of the double line change path is shown in Figure 12. When the reference velocity is set to *Vref* = 40 km/h, the maximum lateral and longitudinal errors using the backward Euler method were 0.3034 m and 0.0203 m, in contrast to 0.3827 m and 0.0412 m using the forward Euler method. The maximum heading errors were 0.0673 rad using the backward Euler method and 0.0648 rad using the forward Euler method. When the reference velocity is set to *Vref* = 60 km/h, the maximum lateral and longitudinal errors using the backward Euler method were 0.587 m and 0.0504 m, in contrast to 0.6187 m and 0.0311 m using the forward Euler method. The maximum heading errors were 0.1035 rad using the backward Euler method and 0.0967 rad using the forward Euler method. In sum, the MPC controller using the backward Euler method had a better tracking accuracy in the circular path than that using the forward Euler method. Figure 13 and Figure 14 show the articulated acceleration and steer angle, respectively.

**Figure 15:**
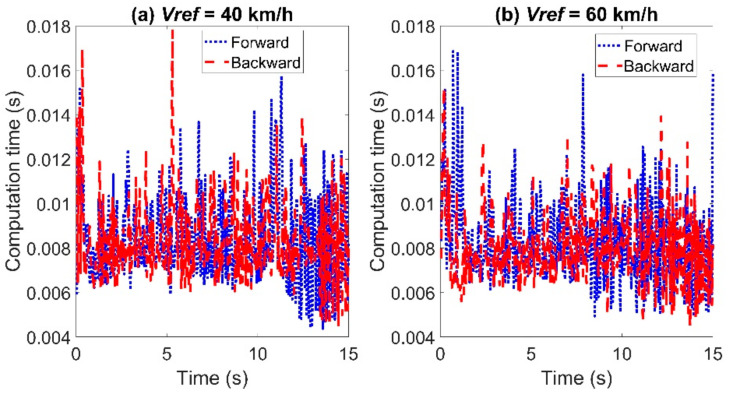
Comparison of the computation time for tracking the double line change path with *T_s_* = 0.05 s: left for Case (**a**) with *Vref* = 40 km/h and right for Case (**b**) with *Vref* = 60 km/h.

The comparison of the calculation time between the two controllers is shown in Figure 15. When the reference velocity is set to *Vref* = 40 km/h, the maximum calculation time using the backward Euler method was 0.0178 s, and the average calculation time was 0.0084 s, in contrast to 0.0157 s and 0.0084 s using the forward Euler method. When the reference velocity is set to *Vref* = 60 km/h, the maximum calculation time using the backward Euler method was 0.0152 s and the average calculation time was 0.008 s, in contrast to 0.0169 s and 0.0083 s using the forward Euler method.

Through three sets of comparisons, we can draw some conclusions. First, the lateral tracking errors using the backward Euler method were much smaller than those when using the forward Euler method. Second, the lateral tracking errors using either the forward or backward Euler method increased with the reference velocity. Third, the calculation times using the backward Euler method were almost the same with that using the forward Euler method. Lastly, compared with the articulated acceleration, there was a clear discrepancy in the articulated steer angle.

## 5. Conclusions

An effective and efficient method for generating a feasible trajectory is of vital importance to meet the requirement of instantaneous control for autonomous driving. In this paper, we have proposed a trajectory tracking controller based on MPC. Most MPC-based and other methods either set the velocity to a constant or cannot actively adjust the longitudinal velocity according to the information of the reference trajectory. To solve this problem, both the acceleration and steer angle are set to control inputs. Hence, the proposed controller can automatically adjust the velocity according to the information of the reference trajectory. Moreover, instead of the forward Euler integration method, the backward Euler integration method is used to establish the predictive model. To meet the real-time requirement, we impose the constraints *u_k+_*_1_ = … = *u_k+Nc_* on the control law. This significantly reduced the problem complexity. The warm-start technique was used to further accelerate the convergence of the optimization solver of the controller by using the previous results as a guess for the current optimization problem.

The proposed closed-loop MPC controller was stable and validated by simulation experiments. Compared with the MPC controller using the forward Euler method, the MPC controller using the backward Euler method had a much better accuracy in the lateral error, which is an important indicator to ensure driving safety. The lateral error could be reduced by up to 78%. There is little difference in the longitudinal error between the two controllers. However, the heading error of the MPC controller using the backward Euler method was larger than that of the MPC controller using the forward Euler method. The maximum and average computation times using the backward Euler method were almost the same or slightly larger than those using the forward Euler method. Moreover, the MPC controller using backward Euler method was more robust than that using the forward Euler method. The threshold value of the velocity for the MPC controller using the backward Euler method was larger than that using the forward Euler method (83 km/h versus 67.7 km/h for the sinusoidal trajectory). Overall, the MPC controller using the backward Euler method had a better tracking accuracy at the cost of no or little computation time.

The existence of the discrepancy between the actual trajectory and the reference trajectory is mainly due to the modelling errors, computation errors and disturbance errors. Recent studies on MPC-based controllers mainly focus on the modelling errors and disturbance errors. Few authors investigated the computation errors during the discretization for nonlinear systems. We hope that this paper is instructive and allows researchers new insight into creating MPC-based controllers.

From the perspective of science, our contribution is to give a new way to establish a predictive model, which is a cornerstone of designing a path tracking controller. Besides the forward and backward Euler methods, there are several integration methods, such as the midpoint method and Runge-Kutta methods. Improving the accuracy of the prediction model using other integration methods could be a promising way to get an effective control to maintain a good tracking accuracy.

## Figures and Tables

**Table 1:** Parameters of the vehicle and the controller.

Parameter	Value
*l_f_*	1.232 m
*l_r_*	1.468 m
Range of *a*	[−1 m/s^2^, 1 m/s^2^]
Range of *δ*	[−0.44 rad, 0.44 rad]
Range of lateral error	[−0.5 m, 0.5 m]
*N_P_*	15
*Nc*	1
*Q*	100*I* ^1^
*R*	*I*

^1^ *I* is the unit matrix.

## Data Availability

Not applicable.
